# Construction of community home-based older adult care service model based on modular design concept

**DOI:** 10.3389/fpubh.2025.1672918

**Published:** 2025-10-08

**Authors:** Xiaohua Zhou, Lina Chen, Xu Lou, Ying Li, Bobo Han

**Affiliations:** School of Nursing, Dalian University, Dalian, China

**Keywords:** community home-based older adult care service, demand, supply, modular, model construction, caregiver support

## Abstract

**Objectvie:**

To address the supply–demand mismatch in community home-based older adult care services amid China’s deepening aging population crisis.

**Methods:**

This study employs a modular design concept, selecting Liaoning Province—the region with the nation’s highest aging rate—as the research area. A questionnaire survey was conducted among 331 community-dwelling older adults, and multiple linear regression analysis was applied to identify factors influencing care service demands.

**Results:**

Key findings include: (1) Older adult’ demands manifest a five-dimensional structure (life assistance, medical care, spiritual/cultural engagement, rights protection, and age-friendly modifications), with spiritual/cultural engagement (score rate: 68.40%) and age-friendly modifications (67.67%) being the most urgent needs. (2) Subgroups including advanced age (≥71 years), disabled, living alone, highly educated, and chronic disease individuals exhibited significantly higher demand intensity (*p* < 0.05). For instance, the regression coefficient (*B*) for medical care demand among the disabled reached 0.545. (3) Based on these results, a modular service framework was constructed, featuring five functionally independent core modules. A dynamic service package combination function was innovatively proposed, utilizing a module activation coefficient (*α_k,i_*) and an module weighting factor (*β_k,i_*) to achieve precise customization.

**Conclusion:**

Centered on community residents’ committees (CRCs) as coordination hubs, this model enables dynamic monitoring and optimization through the Demand-Service Matching Index (DSMI), offering an actionable solution to reconcile fragmented resources and heterogeneous demands, thereby supporting both older adults and their caregivers in regional older adult care systems.

## 1 Introduction

Population aging represents a global phenomenon, initially pioneered by developed nations. In recent years, China has experienced a notably accelerated pace of population aging, surpassing both developed countries and the global average (1). According to the 2023 National Report on the Development of Aging Initiatives in China, by the end of 2023, the population aged 60 and above reached 296.97 million, accounting for 21.1% of the total population, while those aged 65 and above stood at 216.76 million (15.4% of the total population). Projections indicate that by mid-century, China’s population aged 60 and above will approach 500 million, constituting 35% of its total population, thereby transitioning China into a super-aged nation confronting profound aging-related challenges (2). Population aging has emerged as a critical challenge for China. Data from China’s Seventh National Population Census reveal that Liaoning Province, with a resident population of 42.59 million, hosts 10.954 million individuals aged 60 and above (25.72% of its population) and 17.42% aged 65 and above. This positions Liaoning as the region with the highest proportion of older population in China, facing unprecedented older adult care pressure. Consequently, this study selects Liaoning Province as its empirical context.

Another distinctive feature of China’s population structure is the concurrent progression of population aging and family miniaturization. The prevalence of “4–2-1” or “4–2-2” family frameworks—where four grandparents and two parents support one or two children—has substantially diminished traditional family-based older adult care capacity. Meanwhile, institutional care services face limitations in development and managerial capabilities, compounded by public skepticism toward institutional models (3). The growing demand for older adult care disproportionately relies on informal care networks, primarily family members, who often operate with limited resources and formal support. This places a significant physical, psychological, and social burden on these caregivers, compromising the quality of care and their own well-being (4, 5).

Community is the fundamental unit of social governance. Community home-based older adult care services are centered around the community, integrating medical and health resources with older adult care services. Through convenient and professional services, they provide continuous and integrated services such as hospitalization, rehabilitation care, and stable living care for older adults (2). By synthesizing the strengths of both family-based and institutional care, community home-based care optimally accommodates seniors’ preference for “aging in place.” It concurrently alleviates familial caregiving burdens, reduces long-term care costs, and mitigates fiscal pressures on governments (6). Consequently, it has emerged as a crucial supplement to family-based care and a primary modality for addressing aging-related challenges, increasingly favored by older adults.

Despite its advantages, current community home-based older adult care services face challenges such as unequal service distribution, limited service scope, and a mismatch between service supply and demand (7, 8), necessitating urgent systemic improvements. Modularization is a design strategy developed to manage complexity and variety; its core principle involves decomposing a complex system into independent, interchangeable functional units (modules) that can be combined to create customized solutions (9). While foundational applications are in engineering and product design (e.g., LEGO®, IKEA®) (10), the concept has gained significant traction in healthcare and long-term care settings. In the healthcare sector, modular design principles have been applied to the development of the ICU Real-Time Informatics System (11), the construction of healthcare value assessment frameworks (12), and the implementation of cross-departmental, cross-domain solutions that systematically address complex challenges such as sustainable economic growth and universal health coverage (13). In the context of long-term care, modular design has been proposed as an effective strategy to address the heterogeneous needs of older adults while enhancing the efficiency and effectiveness of care delivery (14). The modular approach allows for the creation of flexible service architectures that can be efficiently configured to individual needs, thereby promoting system responsiveness while reducing the burden on caregivers through clear, standardized support options. By breaking down complex care needs into manageable service components, modular design enables more precise matching of services to individual requirements, significantly improving resource allocation and caregiver efficiency.

This integration of modular design into long-term care, particularly within community settings, represents a paradigm shift from rigid, one-size-fits-all service models to flexible, person-centered solutions. This approach is especially pertinent in addressing the multifaceted challenges faced by informal caregivers (often family members), who constitute the backbone of older adult care systems in many societies, including China. The relentless physical, emotional, and financial strain on these caregivers frequently leads to burnout, compromising the quality of care for both the older adult and the caregiver’s own well-being (4). Modular design principles offer a promising framework to mitigate these challenges. By deconstructing complex care needs into standardized, manageable service units, the modular approach can reduce the cognitive and logistical burden on caregivers, providing them with a clear menu of support options that can be efficiently configured to meet evolving needs (14). This not only empowers caregivers by enhancing the predictability and accessibility of resources but also fosters a more sustainable care ecosystem. Therefore, applying modular design to community home-based care is not merely an operational improvement but a strategic intervention aimed at supporting the crucial caregiver workforce upon which the system relies.

Communities, as social entities within defined geographical boundaries, exhibit significant heterogeneity and complexity due to variations in demographic composition, regional planning, and economic development. Modular design principles offer distinct advantages in decomposing complex tasks into relatively simple functional units. Their organizational and technical flexibility enables effective adaptation to inter-community variations and complexities, thereby constituting a viable design framework for contemporary community home-based older adult care services and and building a more sustainable ecosystem for both care recipients and caregivers.

Consequently, this research focuses on addressing two pivotal questions: (1) What specific service items do older adults demand in community home-based care? Which factors significantly influence these demands? (2) Can complex older adult care tasks be decomposed into relatively simple functional units with homogeneous attributes? Can communities optimize resource allocation and provide targeted support to caregivers by selectively combining modular units according to the intensity of older adult care demands within the jurisdiction? This study proposes a modular service model designed to address the heterogeneous demands of older adults while also providing structured support to formal and informal caregivers through customizable service bundles. Through an empirical investigation of older adult residents in Liaoning Province, this study resolves these research questions.

## 2 Research on the current status of community home-based older adult care service demand

### 2.1 Questionnaire design and survey methods

#### 2.1.1 Questionnaire design for service demand assessment

Based on a comprehensive review of domestic and international literature (6, 15, 16), analysis of national and local older adult care policies, referencing home- and community-based care service standards and essential requirements (17), and consultation with domain experts, this study developed *the Survey Questionnaire on Current Demand for Community Home-Based Older Adult Care Services*. The questionnaire comprises two sections: basic demographic information and community home-based older adult care service demands. The service demand section encompasses five dimensions with 19 items: life assistance, medical care, spiritual/cultural engagement, rights protection, and age-friendly modifications. All items were measured using a 5-point Likert scale: 1 = Not needed at all, 2 = Slightly needed, 3 = Neutral, 4 = Moderately needed, 5 = Strongly needed. Higher scores indicate stronger demand for the specific service.

#### 2.1.2 Data collection and processing

Liaoning Province, which has the highest level of population aging in China, comprises 14 cities. This study employed a two-stage sampling strategy: four cities (Shenyang, Jinzhou, Dalian, and Anshan) were first selected through random sampling. Within each city, a convenience sampling method was used to recruit adults aged 60 and above in community public spaces as study participants. This study was conducted in accordance with the Helsinki Declaration and was approved by the Ethical Review Committee of Affiliated Zhongshan Hospital Dalian University (Approval No: KY2023-110-1). The sample size was estimated based on the recommendation of including 5–10 times the number of independent variables in the research tool (18), with an additional 20% allowance for potential missing data. Given that the questionnaire contained 19 variables, the required sample size was calculated to be 114 to 228 participants. Questionnaires were distributed on-site with guidance provided for completion, collected subsequently, and checked for completeness of responses. A total of 357 questionnaires were distributed, with 331 valid questionnaires recovered, yielding a valid response rate of 92.72%.

Data analysis was performed using SPSS 26.0 software. Continuous variables not following a normal distribution are expressed as median (interquartile range) [M (P25, P75)]. Group comparisons were conducted using the Mann–Whitney U test (two groups) or Kruskal–Wallis H test (K ≥ 3 groups). Multiple linear regression was employed to identify independent factors influencing demands for community home-based older adult care services. Prior to regression, key assumptions were verified: linearity was assessed via scatterplots, independence of residuals was confirmed with a Durbin-Watson statistic near 2, normality and homoscedasticity of residuals were examined using P–P plots and scatterplots, respectively. Multicollinearity was assessed using variance inflation factors (VIF) and tolerance (T) statistics (VIF < 5 and Tolerance > 0.1 for all variables). Variables with *p* < 0.05 in univariate analyses were included in the multivariate model using the Enter method. A *p*-value below 0.05 was considered statistically significant.

The statistical analysis strategy in this study was designed to provide an empirical foundation for the subsequent construction of the modular service model. Specifically, the significant influencing factors identified by the multiple linear regression analysis and their standardized regression coefficients (*B* values) were intended to be directly used to calculate the individual weighting factors (*β_i_*). Concurrently, the score distribution for each demand dimension (e.g., median and interquartile range) was to inform the determination of thresholds (e.g., the 75th percentile) for the module activation coefficients (*α_k_*). The complete mathematical formulation of the module dynamic combination mechanism, including the service bundle function, and the operational details of the Demand-Service Matching Index (DSMI) are elaborated in Section 3 (Model Construction).

### 2.2 Survey results on the status of community home-based older adult care service demand

#### 2.2.1 Descriptive statistics of service demand items

The demand level for community home-based older adult care services, ranked from highest to lowest based on score rate, was as follows: spiritual/cultural engagement (68.40%), age-friendly modifications (67.67%), life assistance (65.38%), medical care (63.82%), and rights protection (62.92%). The specific scoring situation is shown in Table 1.

**Table 1:** Scores of various items in the demand for community home-based care service among the older adult (*n* = 331).

Service items	[M(P25, P75)]	Service items	[M(P25, P75)]
**Life assistance**	**3.40 (3.00**–**3.80)**	**Spiritual/Cultural engagement**	**3.33 (3.00**–**4.00)**
Hygiene care	3.00 (2.00–4.00)	Spiritual comfort	3.00 (3.00–4.00)
Mobility assistance	3.00 (2.00–3.00)	Leisure activities	4.00 (3.00–4.00)
Catering services	3.00 (3.00–4.00)	Self-actualization	3.00 (3.00–4.00)
Entrusted agency services	3.00 (3.00–4.00)	**Rights protection**	**3.00 (2.67**–**4.00)**
Emergency maintenance	4.00 (3.00–5.00)	Conflict mediation	3.00 (3.00–4.00)
**Medical care**	**3.17 (2.67**–**3.83)**	Legal consultation/aid	3.00 (2.00–4.00)
Health management	4.00 (3.00–4.00)	Policy service promotion	3.00 (2.00–4.00)
Preventive healthcare	3.00 (3.00–4.00)	**Age-friendly modifications**	**3.50 (3.00**–**4.00)**
Diagnosis & treatment	3.00 (3.00–4.00)	Indoor modifications	3.00 (2.00–4.00)
Rehabilitation guidance	3.00 (2.00–4.00)	Public environment modifications	3.00 (3.00–4.00)
Nursing care	3.00 (2.00–4.00)		
Emergency rescue	3.00 (2.00–4.00)		

Bold font indicates the average scores across the five dimensions.

#### 2.2.2 Univariate analysis of demand across subgroups

The results of the univariate analysis, comparing demand scores across different demographic and health subgroups using non-parametric tests (Mann–Whitney U or Kruskal-Wallis H test), are summarized in Table 2. Significant differences (*p* < 0.05) in demand were observed based on age, education level, spouse status, living arrangement, self-care ability, and the presence of chronic diseases. ① Age: Participants were categorized into four age groups: 60–65 years (108, 32.63%), 66–70 years (74, 22.36%), 71–80 years (110, 33.23%), and ≥81 years (39, 11.78%). Overall, demand for community home-based older adult care services increased significantly with age. Notably, older adults aged ≥71 years exhibited significantly higher demand across all service categories. ② Education level: Participants were divided into two groups based on educational attainment: junior high school or below (250, 75.53%), and senior high school or above (81, 24.47%). Older adults with higher education levels demonstrated greater demand for services. ③ Spouse: Individuals who were never married, divorced, or widowed were defined as “without a spouse.” Those without a spouse (94, 28.40%) exhibited higher demand for all services compared to those with a spouse (237, 71.60%). ④ Number of children: The distribution of participants by number of children was: 0 children (9/331, 2.7%), 1 child (148/331, 44.7%), 2 children (112/331, 33.8%), 3 children (43/331, 13.0%), and 4 children (19/331, 5.7%). Participants were grouped as having ≤1 child or ≥2 children to assess the impact on service demand. Results indicated that older adults with fewer children relied more heavily on medical care and spiritual/cultural engagement compared to those with more children. ⑤ Living alone: Older adults living alone (57, 17.22%) exhibited significantly stronger demand for community home-based older adult care services than non solitary older adults (274, 82.78%), with significantly higher demand scores across all service categories (all *p* < 0.001). ⑥ Self-care ability: Define older adults who are partially or completely unable to take care of themselves as disabled older adults. The results showed that the demand score of disabled older adults (65, 19.64%) was significantly higher than that of fully independent individuals (266, 80.36%; *p* < 0.01), especially in terms of life assistance and medical care services, where the difference was the greatest. ⑦ Income levels: Primary income sources for participants were pensions and retirement benefits. Monthly income was categorized into four levels: 0–1,500 yuan (28, 8.46%), 1,501–3,000 yuan (138, 41.69%), 3,001–4,999 yuan (113, 34.14%), and >5,000 yuan (52, 15.71%). Significant between-group differences were only found for demand related to age-friendly modifications (*p* = 0.037), with individuals earning ≤1,500 yuan per month expressing the highest demand. ⑧ Chronic diseases: Participants were dichotomized based on the presence of chronic diseases (e.g., hypertension, coronary heart disease, diabetes, rheumatism). Compared to those without chronic diseases (96, 29.00%), individuals with chronic conditions (235, 71.00%) exhibited significantly higher demand for life assistance (*p* < 0.001), medical care (*p* < 0.001), rights protection (*p* = 0.024), and age-friendly modifications (*p* = 0.003).

**Table 2:** Comparison of demand for community home-based older adult care services for older adults with different characteristics [M (P25, P75)].

Characteristic		Service items				
		Life assistance	Medical care	Spiritual/Cultural engagement	Rights protection	Age-friendly modifications
Age group (years)	60–65	3.00 (2.65–3.40)	3.17 (2.33–3.17)	3.33 (3.00–3.92)	3.00 (3.00–3.92)	3.00 (3.00–4.00)
	66–70	3.10 (2.75–3.45)	3.00 (2.13–3.33)	3.00 (2.00–4.00)	3.00 (2.00–3.33)	3.00 (2.00–4.00)
	71–80	3.40 (3.00–4.00)	3.17 (2.83–4.00)	4.00 (3.00–4.33)	3.00 (3.00–4.00)	4.00 (3.00–4.50)
	≥81	3.80 (3.40–4.00)	3.50 (3.17–4.00)	4.00 (2.33–4.00)	3.00 (2.33–4.00)	4.00 (2.50–4.50)
	*F* value	44.849	33.935	18.094	12.137	20.487
	*p* value	0.000*	0.000*	0.000*	0.007*	0.000*
Education level	Junior high school or below	3.30 (2.80–3.60)	3.17 (2.50–3.54)	3.33 (3.00–4.00)	3.00 (2.00–3.67)	3.50 (2.88–4.00)
	Senior high school or above	3.40 (3.00–4.00)	3.50 (3.00–4.00)	3.67 (3.00–4.67)	3.33 (3.00–4.67)	4.00 (3.00–5.00)
	*Z* value	−2.244	−3.472	−3.21	−4.57	−2.16
	*p* value	0.025*	0.001*	0.000*	0.000*	0.030*
Spouse	None	3.80 (3.40–4.00)	4.00 (3.17–4.17)	4.00 (3.33–4.67)	3.67 (3.00–4.00)	4.00 (3.50–4.50)
	Yes	3.20 (2.80–3.40)	3.00 (2.33–3.33)	3.33 (3.00–4.00)	3.00 (2.00–3.33)	3.00 (2.75–4.00)
	*Z* value	−7.688	−7.917	−5.720	−4.397	−5.814
	*p* value	0.000*	0.000*	0.000*	0.000*	0.000*
Number of children	≤1	3.40 (3.00–3.80)	3.17 (3.00–3.83)	3.67 (3.00–4.00)	3.00 (3.00–4.00)	3.50 (3.00–4.00)
	≥2	3.40 (2.80–3.60)	3.17 (2.33–3.83)	3.33 (2.33–4.00)	3.00 (2.00–3.33)	3.50 (2.00–4.00)
	*Z* value	−0.728	−2.557	−3.262	−4.632	−1.805
	*p* value	0.467	0.011*	0.001*	0.000*	0.071
Living arrangement	Living alone	4.00 (3.50–4.20)	4.00 (3.17–4.58)	4.00 (4.00–4.83)	4.00 (3.00–4.00)	4.00 (4.00–4.50)
	Not living alone	3.20 (2.80–3.40)	3.00 (2.33–3.50)	3.33 (2.67–4.00)	3.00 (2.00–3.67)	3.00 (2.50–4.00)
	*Z* value	−7.493	−6.930	−6.569	−5.329	−4.715
	*p* value	0.000*	0.000*	0.000*	0.000*	0.000*
Self-care ability	Fully independent	3.20 (2.80–3.40)	3.00 (2.33–3.33)	3.33 (3.00–4.00)	3.00 (2.33–3.67)	3.00 (3.86–4.00)
	Disabled	4.00 (3.60–4.20)	4.00 (3.50–4.67)	4.00 (3.00–5.00)	4.00 (3.00–4.67)	4.00 (4.00–5.00)
	*Z* value	−8.121	−8.119	−4.179	−4.544	−6.265
	*p* value	0.000*	0.000*	0.009*	0.002*	0.000*
Income levels (yuan/month)	0–1,500	3.40 (3.20–4.00)	3.17 (2.71–4.08)	4.00 (3.08–4.92)	3.67 (2.67–3.92)	4.00 (3.13–4.50)
	1,501–3,000	3.20 (2.80–3.60)	3.08 (2.33–4.00)	3.33 (3.00–4.00)	3.00 (2.00–4.00)	3.00 (2.00–4.00)
	3,001–4,999	3.40 (3.00–3.80)	3.17 (3.00–4.00)	3.33 (3.00–4.00)	3.00 (3.00–3.83)	3.50 (3.00–4.00)
	>5,000	3.40 (3.00–3.75)	3.17 (2.63–3.50)	3.33 (3.00–4.00)	3.00 (2.67–4.00)	3.00 (2.00–4.00)
	*F* value	6.574	5.312	7.635	3.034	8.481
	*p* value	0.087	0.150	0.054	0.386	0.037*
Chronic diseases	None	3.00 (2.45–3.55)	2.83 (2.21–3.33)	3.67 (2.33–4.00)	3.00 (2.00–4.00)	3.00 (2.00–4.00)
	Yes	3.40 (3.00–3.80)	3.17 (3.00–4.00)	3.33 (3.00–4.00)	3.00 (3.00–4.00)	4.00(3.00–4.00)
	*Z* value	−3.516	−4.411	−0.202	−2.250	−2.930
	*p* value	0.000*	0.000*	0.840	0.024*	0.003*

*p<0.05.

#### 2.2.3 Multivariate analysis of influencing factors

To identify independent factors influencing demand, multiple linear regression analyses were performed for each of the five demand dimensions. The results are presented in Table 3. The assumptions of linear regression were verified prior to analysis (as detailed in Section 2.1.2). Variables such as advanced age, disability, living alone, and higher education level were significant predictors of higher demand across multiple service modules. ① Life assistance: Regression analysis identified age, education level, spouse, living arrangement, self-care ability, and chronic disease as independent significant factors influencing demand for life assistance (all *p* < 0.05). Specifically: Demand was significantly higher among older adults aged 71–80 (*B* = 0.215, *p* = 0.013) and ≥81 years (*B* = 0.287, *p* = 0.037) compared to those aged 60–65. Individuals with a higher education levels exhibited greater demand (*B* = 0.265, *p* = 0.001). Demand was significantly higher among those without a spouse (*B* = 0.206, *p* = 0.037) and those living alone (*B* = 0.381, *p* = 0.001). Disabled older adults showed the most pronounced increase in demand (*B* = 0.403, *p* < 0.001). ② Medical care services: Demand for medical care services was significantly influenced by age, education level, spouse, living arrangement, self-care ability, chronic disease, and number of children (all *p* < 0.05). Individuals aged 71–80 years reported higher demand (*B* = 0.258, *p* = 0.008), while the ≥81 years group showed no significant difference (*p* = 0.324) compared to 60–65 years. Those with a higher education levels placed greater emphasis on medical care (*B* = 0.399, *p* < 0.001). Individuals with ≥2 children exhibited lower demand (*B* = −0.207, *p* = 0.010). Demand was significantly higher among those with chronic diseases (*B* = 0.253, *p* = 0.003) and disabled (*B* = 0.545, *p* < 0.001). ③ Spiritual/cultural engagement: Age, education level, living arrangement, and number of children significantly impacted demand for spiritual/cultural engagement. Demand was higher in the 71–80 years group (*B* = 0.344, *p* = 0.004). Individuals with a higher education levels (*B* = 0.379, *p* = 0.001) and those living alone (*B* = 0.533, *p* = 0.001) reported significantly greater demand. Individuals with ≥2 children exhibited lower demand (*B* = −0.249, *p* = 0.012). ④ Rights protection: Education level, self-care ability, and number of children were the primary factors influencing demand for rights protection. Individuals with a a higher education levels demonstrated greater concern for rights protection (*B* = 0.512, *p* < 0.001). Disabled older adults showed higher demand for rights protection (*B* = 0.551, *p* < 0.001). ⑤ Age-friendly modifications: Low-income and disabled groups exhibited the most urgent need for age-friendly modifications. Individuals with an income >5,000 yuan reported significantly lower demand (*B* = −0.726, *p* = 0.001), while the low-income group (0–1,500 yuan) showed the highest demand, reflecting the constraints imposed by economic conditions on housing modifications. Disabled individuals exhibited the strongest demand intensity (*B* = 0.697, *p* < 0.001).

**Table 3:** Regression analysis results of demand for community home-based older adult care services.

Dimension	Variables		Non standardized coefficient		Standardized Coefficient	*t*	Significance	Collinearity statistics	
			*B*	Standard error	Beta			Tolerance	VIF
Life assistance	Constant		2.789	0.082		34.126	0		
	Age (year)	66–70	−0.062	0.092	−0.036	−0.678	0.498	0.746	1.341
		71–80	0.215	0.087	0.14	2.488	0.013*	0.654	1.528
		≥81	0.287	0.137	0.128	2.098	0.037*	0.558	1.792
		60–65	0						
	Education level	Senior high school or above	0.265	0.081	0.157	3.266	0.001*	0.897	1.115
		Junior high school or below	0						
	Spouse	None	0.206	0.098	0.128	2.095	0.037*	0.553	1.808
		Yes	0						
	Living arrangement	Living alone	0.381	0.115	0.198	3.301	0.001*	0.575	1.74
		Not living alone	0						
	Self-care ability	Disabled	0.403	0.111	0.221	3.642	0.000*	0.565	1.771
		Fully independent	0						
	Chronic diseases	Yes	0.17	0.076	0.106	2.24	0.026*	0.922	1.084
		None	0						
Medical care	Constant		2.707	0.099		27.478	0		
	Age (year)	66–70	−0.167	0.101	−0.083	−1.647	0.101	0.736	1.359
		71–80	0.258	0.097	0.145	2.667	0.008*	0.633	1.579
		≥81	0.151	0.153	0.058	0.988	0.324	0.541	1.85
		60–65	0						
	Education level	Senior high school or above	0.399	0.093	0.205	4.284	0.000*	0.82	1.22
		Junior high school or below	0						
	Spouse	None	0.326	0.108	0.176	3.012	0.003*	0.553	1.809
		Yes	0						
	Living arrangement	Living alone	0.294	0.128	0.133	2.296	0.022*	0.564	1.772
		Not living alone	0						
	Self-care ability	Disabled	0.545	0.121	0.259	4.486	0.000*	0.565	1.771
		Fully independent	0						
	Chronic diseases	Yes	0.253	0.083	0.137	3.036	0.003*	0.922	1.084
		None	0						
	Number of children	≥2 children	−0.207	0.079	−0.124	−2.608	0.010*	0.835	1.197
		1 child	0						
Spiritual/Cultural engagement	Constant		3.202	0.098		32.808	0		
	Age (year)	66–70	−0.15	0.125	−0.068	−1.198	0.232	0.736	1.358
		71–80	0.344	0.119	0.177	2.879	0.004*	0.635	1.574
		≥81	0.045	0.188	0.016	0.241	0.810	0.545	1.836
		60–65	0						
	Education level	Senior high school or above	0.379	0.114	0.178	3.327	0.001*	0.837	1.195
		Junior high school or below	0						
	Spouse	None	0.169	0.133	0.083	1.268	0.206	0.558	1.793
		Yes	0						
	Living arrangement	Living alone	0.533	0.158	0.22	3.375	0.001*	0.565	1.769
		Not living alone	0						
	Self-care ability	Disabled	0.162	0.147	0.070	1.100	0.272	0.588	1.7
		Fully independent	0						
	Number of children	≥2 children	−0.249	0.098	−0.136	−2.538	0.012*	0.835	1.197
		1 child	0						
Rights protection	Constant		2.986	0.122		24.514	0		
	Age (year)	66–70	−0.371	0.125	−0.165	−2.954	0.003*	0.736	1.359
		71–80	0.081	0.12	0.041	0.674	0.501	0.633	1.579
		≥81	−0.465	0.189	−0.161	−2.46	0.014*	0.541	1.850
		60–65	0						
	Education level	Senior high school or above	0.512	0.115	0.236	4.445	0.000*	0.82	1.22
		Junior high school or below	0						
	Spouse	None	0.105	0.134	0.051	0.784	0.434	0.553	1.809
		Yes	0						
	Living arrangement	Living alone	0.33	0.158	0.133	2.087	0.038*	0.564	1.772
		Not living alone	0						
	Self-care ability	Disabled	0.551	0.15	0.234	3.665	0.000*	0.565	1.771
		Fully independent	0						
	Chronic diseases	Yes	0.146	0.103	0.071	1.42	0.156	0.922	1.084
		None	0						
	Number of children	≥2 children	−0.29	0.098	−0.155	−2.949	0.003*	0.835	1.197
		1 child	0						
Age-friendly modifications	Constant		3.42	0.216		15.844	0		
	Age (year)	66–70	−0.414	0.14	−0.168	−2.952	0.003*	0.744	1.344
		71–80	0.055	0.134	0.025	0.412	0.681	0.641	1.561
		≥81	−0.321	0.214	−0.101	−1.498	0.135	0.532	1.879
		60–65	0						
	Education level	Senior high school or above	0.458	0.131	0.192	3.491	0.001*	0.799	1.252
		Junior high school or below	0						
	Spouse	None	0.324	0.151	0.143	2.148	0.032*	0.548	1.825
		Yes	0						
	Living arrangement	Living alone	0.071	0.176	0.026	0.405	0.686	0.574	1.742
		Not living alone	0						
	Self-care ability	Disabled	0.697	0.17	0.27	4.106	0.000*	0.558	1.793
		Fully independent	0						
	Chronic diseases	Yes	0.187	0.117	0.083	1.599	0.111	0.905	1.105
		None	0						
	Income levels (yuan/month)	1,501–3,000	−0.408	0.195	−0.196	−2.093	0.037*	0.275	3.639
		3,001–4,999	−0.368	0.199	−0.17	−1.849	0.065	0.286	3.498
		>5,000	−0.726	0.226	−0.258	−3.214	0.001*	0.376	2.662
		0–1,500	0						

*p < 0.05.

### 2.3 Discussion of factors influencing community home-based older adult care services demands

This study categorized the demand for community home-based older adult care services into five dimensions: life assistance, medical care, spiritual/cultural engagement, rights protection, and age-friendly modifications. Demand within each dimension was evaluated using a Likert 5-point scale, where higher scores indicated stronger demand for that specific category of services. The results revealed that the overall demand for community home-based older adult care services among the older adult is at a medium to low level (with scores ranging from 62.92 to 68.40% across dimensions). This phenomenon may be potentially associated with both traditional Chinese cultural values and the specific characteristics of the older adult sample in this study. On one hand, influenced by Confucian principles emphasizing filial piety, a majority of older adults in China still prefer aging in place and receiving care from family members (19). On the other hand, as the study sample was recruited from public community spaces, functionally dependent individuals or those of advanced age with the most urgent service needs may not have been adequately represented.

#### 2.3.1 Age and self-care ability: key variables for demand differentiation

The demand for life assistance (71–80 years: *B* = 0.215, *p* = 0.013; ≥81 years: *B* = 0.287, *p* = 0.037) and medical care (71–80 years: *B* = 0.258, *p* = 0.008) increased significantly with age, a trend directly associated with declining physical function. Disabled older adults (partially/completely dependent) exhibited significantly higher demand across all service categories (life assistance: *B* = 0.403; medical care: *B* = 0.545; rights protection: *B* = 0.551; age-friendly modifications: *B* = 0.697; all *p* < 0.001), confirming that physical health status imposes a fundamental constraint on essential care needs (20). Among all age groups of older adults, individuals aged 66–70 exhibited relatively lower overall demand for services. This phenomenon may be attributed to two factors: on the one hand, compared to the oldest-old, they are generally healthier, more physically active, and more independent (21); on the other hand, having largely passed the adaptation period following the conventional retirement age of 55–60 in China, this group tends to exhibit greater stability in both psychological and daily living status.

#### 2.3.2 Education level: a moderating factor for demand perception and acceptance

Older adults with a senior high school education or above exhibited significantly higher demand across all service categories. This phenomenon can potentially be explained by several factors: ① Enhanced information access and openness: Individuals with higher education levels typically possess greater capacity for information acquisition and demonstrate greater openness to novel concepts. Community home-based eldercare, as a relatively novel concept in eldercare provision, may be more readily accepted by this group and perceived as capable of meeting their multifaceted needs (22). ② Socioeconomic status and quality-of-life focus: Older adults with higher educational attainment often enjoy relatively higher social status and income levels. Consequently, they tend to place greater emphasis on maintaining quality of life in later years, fulfilling psychosocial and cultural aspirations, and safeguarding their legitimate rights and interests (23).

#### 2.3.3 Living arrangement: highlighting the vulnerability of living alone older adults

Individuals living alone exhibited significantly higher demand for life assistance (*B* = 0.381, *p* = 0.001), medical care (*B* = 0.294, *p* = 0.022), and spiritual/cultural engagement (*B* = 0.533, *p* = 0.001) compared to their non-solo-living counterparts. This model reflects an increased reliance on community services resulting from the absence of familial support structures. A previous study (24) did not find that marital status affects the demand for informal care among older adults. The results of this study, however, indicate that older adults living with a spouse have significantly lower demand for life assistance and medical care. This finding suggests that mutual support between spouses also plays an important role in meeting care needs in later life. Therefore, these findings underscore the critical role of spousal support as a primary buffer against care dependency, highlighting that living arrangement, particularly solitary living, serves as a key indicator for identifying older adults at high risk of requiring comprehensive community-based services.

#### 2.3.4 Economic income: limited overall impact but structural differences exist

Overall, income demonstrated no significant effect on demand for the majority of services (*p* > 0.05). This limited influence may be potentially attributable to the mitigation of financial constraints through widespread medical insurance coverage (25) and local subsidy policies (e.g., senior age allowances). A notable exception emerged concerning demand for age-friendly modifications. The low-income group (0–1,500 yuan) exhibited significantly higher demand compared to the high-income group (>5,000 yuan: *B* = −0.726, *p* = 0.001). This disparity likely reflects poorer housing conditions among low-income individuals, necessitating greater need for environmental adaptations. Furthermore, this result underscores that income differentials exert a more pronounced influence on demand for non-essential services compared to core care needs.

In summary, age, self-care ability, education level, and living arrangement are significant factors influencing the demand for community home-based older adult care services among the older adult. To enhance service delivery, community decision-makers should systematically survey the older adult within their jurisdiction, incorporate key influencing factors such as living arrangement and chronic diseased into an early warning system for demand assessment, and refine the community home-based older adult care service system across multiple dimensions to achieve precise service matching. Establish a demand-based tiered response system, prioritizing disabled individuals, advanced-age seniors (≥71 years), and living alone older adults as key target groups. For advanced-age disabled individuals, provide integrated home-based care combining medical/nursing services with life assistance where necessary. Establish a regular visitation system to enhance psychosocial support for living alone older adults. Tailor Service Promotion & Delivery: Develop pictorial service manuals and appoint community eldercare advisors to improve service awareness and accessibility for less-educated seniors; Offer value-added services such as legal consultations and cultural salons for highly-educated seniors. Foster a tripartite support network connecting “Community-Family-Medical Institutions,” promoting data interoperability. Additionally, foster a “time-banking” mutual support system to incentivize younger seniors to serve their older counterparts, thereby mobilizing the agency of older adults in actively responding to population aging.

#### 2.3.5 Implications for caregiver support

Our findings on the heterogeneity of older adults’ demands have direct implications for alleviating caregiver burden. The significantly higher service needs identified among subgroups such as the disabled, those living alone, and the advanced-aged underscore the intense pressure faced by their caregivers. For instance, the strong demand for life assistance and medical care among the disabled older adults (*B* = 0.403 and 0.545, respectively) aligns with existing literature (20) highlighting the elevated physical and psychological strain on caregivers supporting individuals with high dependency. The modular service model proposed in section 3 of this study directly addresses this issue by providing structured and standardized support bundles. By providing a clear framework for what services are needed and how they can be combined, the model acts as a decision-support tool, guiding caregivers and service providers toward more effective and comprehensive care plans. This structured approach not only mitigates the overwhelming uncertainty often experienced by family caregivers but also empowers them by enhancing the predictability and accessibility of resources, thereby contributing to a more sustainable care ecosystem.

## 3 Construction of a modular community home-based older adult care service model

### 3.1 Theoretical foundation of the modular design framework

**Figure 1:**
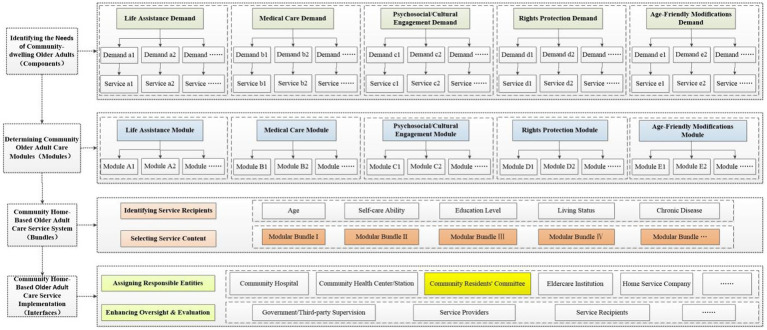
Modular design framework for community home based older adult care services.

Modular design is a methodology that decomposes complex systems into independent, interchangeable functional units. Its core elements comprise: components (the smallest functional units), modules (clusters of components), interfaces (mechanisms for inter-module collaboration), and bundles (customized combinations of modules) (14). Within community home-based older adult care services, service items fulfilling specific older adult needs (such as hygiene care and rehabilitation guidance) can be regarded as components; clusters of functionally related services (such as the life assistance module and the medical care module) can be viewed as modules. Inter-module interfaces facilitate collaboration through service referrals, supervision, evaluation, and other mechanisms among responsible entities. Among these, the Community residents’ committees (CRCs), as a grassroots mass self-governance organization, plays a crucial role in organizing resident self-governance, coordinating community affairs, providing public services, liaising with government agencies, and promoting community development. It serves as a pivotal hub connecting various responsible entities and plays a key role in service oversight and evaluation. Bundles constitute customized service combination plans tailored to the needs of specific target groups (such as living alone older adults or disabled older adults). Based on the aforementioned core elements of modular design, this study constructed a modular design framework diagram applicable to community home-based older adult care services, as shown in Figure 1.

### 3.2 Division and functional definition of core service modules

Based on demand dimension clustering (Table 1) and regression analysis (Table 3), five core functional modules were constructed (Table 4).

**Table 4:** Core modules and functional definitions of community home-based older adult care services.

Module	Core components	Target population (evidence source)	Functional boundary
Life Assistance Module	Hygiene care, Catering services, Emergency maintenance, Entrusted agency services	Age ≥71 years (*B* = 0.215), Disabled (*B* = 0.403), Living alone (=0.381)	Provides daily living support and resolves basic life obstacles
Medical Care Module	Health management, Preventive healthcare, Diagnosis & treatment, Emergency rescue, Rehabilitation guidance	Living alone (*B* = 0.294), Chronic disease (*B* = 0.253), disabled (*B* = 0.545)	Integrates basic medical and nursing services to reduce acute health risks
Spiritual/Cultural Engagement Module	Spiritual comfort, Leisure activities, Self-actualization	Senior high school education or above (*B* = 0.379), Living alone (*B* = 0.533)	Promotes social participation, alleviates loneliness, and enhances psychological well-being
Rights Protection Module	Legal consultation/aid, Policy service promotion, Conflict mediation	Senior high school education or above (*B* = 0.512), Living alone (*B* = 0.33), Disabled (*B* = 0.551)	Safeguards the legitimate rights and interests of older adults
Age-Friendly Modifications Module	Indoor modifications, Public environment modifications	Low-income (0–1,500 yuan/month), Disabled (*B* = 0.697)	Improves residential safety and prevents environmental hazards

### 3.3 Module dynamic combination mechanism

#### 3.3.1 Mathematical representation of service bundles

Define the module combination function, i.e., the service bundle generation formula:


$$S_{i}=\sum_{k=1}^{5}{{\alpha_{k}}_{,}}_{i}\cdot {{\beta_{k}}_{,}}_{i}\cdot M_{k}$$

*S_i_*: Customized service bundle for the *i-th* older adult.

*M_k_*: The *k-th* core module (*k* = 1, 2,… 5).

*α_k,i_*: Module activation coefficient. A binary variable that determines whether the *k-th* service module is activated for the *i-th* older adult. Its value is determined by the following rule: the individual’s demand score is compared to the 75th percentile (P75) threshold of the module’s demand score distribution, which is calculated based on the entire sample (*n* = 331). If the individual’s score ≥ the P75 threshold, then *α_k,i_* = 1, activating the module; otherwise, *α_k,i_* = 0.

**β*_k,i_*: Module Weighting Factor. A continuous variable representing the relative demand intensity of the *i-th* older adult for the *k-th* module. Its value is derived from the sum of the standardized regression coefficients (*B* values) of the significant variables influencing the demand for that specific module. The *β* value for each module is calculated only by summing the *B* values of the variables that have a statistically significant impact on that module, as identified in Table 3.

Application Example: Grandmother Zhang, aged 82, with diabetes and hypertension, widowed, living alone, and disabled. Demand assessment scores: Life assistance = 4.2, Medical care = 4.2, Spiritual/Cultural engagement = 3.5, Rights protection = 3.5, Age-friendly modifications = 4.0. Customize a modular community home-based older adult care service package for this older adult as follows:

(1) Life assistance module.

*α_Life Assistance_*, *_Grandmother Zhang_* = 1 (Demand score 4.2 > Threshold 3.8);

*β_Life Assistance_*, *_Grandmother Zhang_* = 0.287 (Age) + 0.206 (Without a spouse) + 0.381 (Living alone) + 0.403 (Disabled) = 1.277.

(2) Medical care module.

*α_Medical Care, Grandmother Zhang_* = 1 (Demand score 4.2 > Threshold 3.83);

*β_Medical Care, Grandmother Zhang_* = 0.326 (Without a spouse) + 0.294 (Living alone) + 0.545 (Disabled) + 0.253 (Chronic disease) = 1.418.

(3) Spiritual/Cultural engagement module.

*α_Spiritual/Cultural Engagement_*, *_Grandmother Zhang_* = 0 (Score 3.5 < Threshold 4.0).

(4) Rights protection module.

*α_Rights Protection, Grandmother Zhang_* = 0 (Score 3.5 < Threshold 4.0).

(5) Age-friendly modifications module.

*α_Age-Friendly Modifications, Grandmother Zhang_* = 1 (Score 4.0 = Threshold 4.0).

*β_Age-Friendly Modifications_*, *_Grandmother Zhang_* = 0.324 (Without a spouse) + 0.697 (Disabled) = 1.021.

Based on the above analysis, the customized service bundle for Grandma Zhang is as follows:

*S_Grandma Zhang_* = (1 × Life assistance module) × 1.277 + (1 × Medical care module) × 1.418 + (0 × Spiritual/Cultural engagement module) + (0 × Rights protection module) + (1 × Age-friendly modifications module) × 1.021.

#### 3.3.2 Dynamic monitoring and feedback

A Demand-Service Match Index (DSMI) is established to automatically optimize module combinations.


$$\text{DSMI}=\frac{\text{Actual service coverage rate}}{\text{Expected demand coverage rete}}\times \text{Service Satisfaction}$$

Module recombination was automatically triggered when the DSMI value fell at or below 0.8. This threshold was set empirically to indicate a significant misalignment between service provision and measured demand, prompting a revision of the activated modules or their weighting to improve service-person match.

### 3.4 Implementation safeguards for the modular community home-based older adult care service model

#### 3.4.1 Accurate demand identification and dynamic adaptation of service bundles

The provision of community home-based older adult care services must align with the service expectations and priority needs of the older adult within the community. Results from Section 2: Research on the current status of community home-based older adult care service demand in this study indicate that age, disabled, living alone, and chronic disease are core influencing factors on older adults’ demand for these services. Service provision must therefore use these core factors as decision anchors. Based on the results of the multivariate regression analysis of the 331 older adults in this study, advanced age (≥71 years), living alone, and disabled individuals constitute Tier-1 Priority Recipients. The service bundle function is employed to determine activated modules and their respective weighting coefficients (where *α_k,i_* is determined by comparing the demand score to the 75th percentile, and *β_k,i_* is obtained through standardized calculation of the regression coefficient *B* values). Module reconfiguration or service upgrade is triggered when DSMI ≤ 0.8. Leveraging the community older adult demographic profiling database, the service bundle matching values are regularly updated to ensure resource allocation aligns with demand intensity.

#### 3.4.2 Collaborative governance by multiple stakeholders

To achieve the Chinese government’s goal of providing older adult care with security, happiness, and fulfillment, it is necessary to establish a multi-dimensional service system that covers life assistance, medical care, psychosocial support, and other domains. Given the inherent limitations of any single entity in independently providing comprehensive community home-based older adult care services, collaborative governance involving multiple stakeholders has become an essential paradigm for achieving effective service delivery (5, 26). Within this framework: ① The government plays a leading role (27), undertaking responsibilities for strategic planning, resource investment (including funding and personnel training), establishing modular service standards, and fostering a supportive ecosystem for diverse service providers. ② Service providers and module executors (e.g., medical institutions, home service companies, social work organizations) must clearly define their service content and delivery methods. ③ A formal tripartite agreement is established among the government, service providers, and older adult service recipients to clarify rights and responsibilities and implement the collaborative governance model. ④ The Community Residents’ Committee (CRC), leveraging its comprehensive grasp of demographic profiles of the older adult, medical services, home care services, and other integrated information within its jurisdiction, serves as a coordinating hub and exercises oversight functions.

The successful implementation of this modular model hinges on seamless collaboration between the community care system and family caregivers. The model is not designed to replace familial support but to augment it. For instance, the ‘Life Assistance Module’ can provide respite care services, offering temporary relief to family carers, while training programs can be organized to enhance their caregiving capabilities. Furthermore, the Community Residents’ Committee (CRC), serving as the central hub, should establish formal communication channels with families to facilitate information sharing (e.g., through regular caregiver meetings or digital platforms), ensuring care plans are coordinated and that the modules effectively complement the care provided by families.

To translate the modular framework into sustainable practice, each module requires a clear implementation pathway. For example, the Medical Care Module could be operationalized through formal partnerships between CRCs and local primary health centers, specifying protocols for regular health screenings, emergency response, and chronic disease management. Similarly, the Life Assistance Module could be delivered by vetted and trained home service companies or community volunteer organizations, whose service standards and pricing are regulated and made transparent through the smart platform. This detailed delineation of responsibilities and operational workflows ensures that the modular design moves beyond a theoretical concept to an actionable, collaborative service delivery mechanism.

#### 3.4.3 Refining the oversight and evaluation mechanism

Establishing a comprehensive evaluation indicator system is the primary basis for ensuring the quality of eldercare services. It enables service quality to be institutionalized and standardized in a concrete and explicit manner, facilitating implementation and assessment (26). To drive continuous improvement, a three-tiered evaluation chain can be established, encompassing governmental/third-party supervision, self-assessment by service providers, and feedback from end-users. This chain should be supported by a fully open, smart online interactive information platform—leveraging internet technology and led by the government—with support from community offline platforms. This platform enables the responsible parties of various service modules to publish service content and pricing, and to conduct self-evaluations. Concurrently, older adult recipients provide satisfaction feedback through the platform after service consumption. The government or third-party regulatory agencies are responsible for full-process supervision based on this integrated information. Crucially, for any module with a Demand-Service Match Index (DSMI) score of ≤ 0.8, the responsible parties are required to identify the causes and implement corrective improvements. This entire process, from evaluation and feedback to mandated correction, forms a closed-loop quality monitoring system.

#### 3.4.4 Supporting caregivers through modular service delivery

Beyond its direct benefits to older adults, the successful implementation and sustainability of the modular model fundamentally depend on its capacity to support the formal and informal caregivers upon whom the system relies. This model is explicitly designed to function as a supportive framework that reduces the uncertainty and burden often experienced by family caregivers. For instance, the life assistance module can be configured to include respite care services, offering temporary relief to family carers, while training programs can be organized to enhance their caregiving capabilities. Concurrently, the medical care module integrates professional health resources, reducing the family’s direct medical care burden and associated stress. Furthermore, the central coordinating role of the Community Residents’ Committee (CRC) ensures that caregivers have a clear and reliable point of contact for service coordination, information, and psychosocial support. This structured approach not only empowers caregivers by enhancing the predictability and accessibility of services but also fosters a more sustainable care ecosystem by preventing caregiver burnout and promoting the well-being of those providing care.

## 4 Limitations

This study analyzed the service demands of community-dwelling older adults and their influencing factors through empirical investigation, proposing a modular community home-based older adult care service model grounded in modular design principles. This offers a novel paradigm for addressing the “supply–demand mismatch” in such services and providing structured support to caregivers. However, several limitations warrant acknowledgment: First, the convenience sampling method may underrepresent frail, homebound, or cognitively impaired older adults who are less likely to frequent public community spaces. Consequently, the expressed demand levels in this study might be a conservative estimate, as the most vulnerable seniors with the highest care needs are potentially omitted. To address this sampling bias in future research and practical implementation, proactive strategies such as collaboration with community health stations for home-visit assessments or targeted recruitment through neighborhood registries of vulnerable elders are recommended to ensure a more comprehensive representation of the older adult. Second, the data relied on self-reported measures, which are susceptible to social desirability bias. Older adults, particularly those with lower education levels, might underreport their needs due to a desire to be perceived as self-reliant, a tendency to normalize their hardships, or a lack of awareness that certain services could be available. This could further contribute to an underestimation of true demand, especially in psychosocial and rights protection domains. Future studies could combine quantitative surveys with qualitative in-depth interviews to better uncover latent needs and mitigate this bias. Third, the proposed modular service bundle algorithm and dynamic adaptation mechanism (e.g., *S_i_*, DSMI index, weighting factor *β*) lack empirical validation. Future research must prioritize pilot testing this model in diverse community settings. Such pilots are essential to evaluate its practicality, identify potential barriers related to financial constraints, workforce shortages, and policy variability, and iteratively refine the framework for real-world application. Finally, although we stratified age into four groups, the category of ≥81 years encompasses a highly heterogeneous population with vastly different levels of vitality and need. A more granular age classification (e.g., 81–85, 86–90, 90+) in studies with a larger sample size would provide deeper insights into the evolving priorities of the “oldest-old” and allow for even more precise module customization.

Despite these limitations, this study constructs a modular service model that responds to the dual challenge of meeting the heterogeneous demands of community-dwelling older adults while simultaneously providing critical support to their caregivers. By translating complex care needs into a flexible architecture of service modules, our approach offers a pragmatic pathway to mitigate the caregiver burden-a central concern of this research topic. The model’s emphasis on dynamic adaptation and multi-stakeholder collaboration, centered on the Community Residents’ Committee (CRC), provides a scalable framework for communities seeking to build more resilient and sustainable care ecosystems that support both older adults and those who care for them.

## Data Availability

The original contributions presented in the study are included in the article/supplementary material, further inquiries can be directed to the corresponding author.
